# Interrelationships and Methods for Improving University Students' Sense of Gain, Sense of Security, and Happiness

**DOI:** 10.3389/fpsyg.2021.729400

**Published:** 2021-09-24

**Authors:** Linlin Feng, Hao Zhong

**Affiliations:** School of Marxism, Shandong University of Technology, Zibo, China

**Keywords:** sense of gain, sense of security, happiness, prosocial behaviors, university students

## Abstract

The report of the 19th National Congress of the Communist Party of China stressed the necessity to “keep up with people's ever-growing needs for a better life. We will continue to promote social fairness and justice, develop effective social governance, and maintain public order. With this we should see that our people will always have a strong sense of gain, happiness, and sense of security.” In this study, 646 university students were surveyed using the Demographic Questionnaire, Sense of Gain Scale, Sense of Security Scale, Orientations to Happiness Scale, and Prosocial Behavior Tendencies Scale to explore the relationships among sense of gain, sense of security and happiness (including meaning, pleasure, and engagement happiness), and to discuss methods for enhancing them on the basis of prosocial behaviors. The results revealed that (1) sense of gain had significant positive correlations with meaning, pleasure, and engagement happiness but a significant negative correlation with sense of security. Sense of security did not have a significant correlation with the three types of happiness. Prosocial behaviors had significant positive correlations with sense of gain and the three types of happiness but shared no significant correlation with sense of security. (2) Sense of gain significantly and positively predicted meaning, pleasure, and engagement happiness, whereas the interaction between sense of security and sense of gain did not yield a significant prediction for the three types of happiness. Prosocial behaviors significantly and positively predicted sense of gain and the three types of happiness. (3) Sense of gain had mediating effects on the relationships between prosocial behaviors and meaning, pleasure, and engagement happiness, whereas sense of security did not have a moderating effect on the relationships between sense of gain and the three types of happiness. Prosocial behaviors directly affect meaning, pleasure, and engagement happiness and can indirectly influence happiness through enhancing a sense of gain. The implementation of prosocial behaviors can not only provide help for others, but also promote the self-satisfaction of the behavior agents and help them get more happiness.

## Introduction

General Secretary Xi Jinping proposed the concepts of sense of gain, happiness, and sense of security of the people for the first time at the 19th National Congress of the Communist Party of China, emphasizing the need to “strive to ensure and improve living standards and make steady progress toward enhancing our people's sense of gain, happiness, and sense of security and toward realizing common prosperity for everyone” (Wen, 2017). The meaning of sense of gain, happiness, and sense of security is the subjective perception of the state of real life. Sense of gain is the subjective feeling based on an individual's acquisition of actual benefits in comparison with their expected acquired benefits. Sense of gain refers to the degree to which the subjective feeling resulting from the actual benefits obtained through labor is consistent with the expected labor value. Happiness refers to the subjective feeling produced after an individual compares their own expectations of life with the actual state of life, and is the subjective feeling generated when the perceived need for a more favorable life is consistent with the individual's actual state of life. Sense of security is an individual's subjective affirmation of having a stable and peaceful life and refers to the belief that one's own safety of life and property, and other interests are guaranteed and supported. When encountering a risk, the individual believes themselves or society to possess sufficient capacity to mitigate the risk (Jin and Tao, 2018; Wang and Liu, 2019).

Although the levels and emphases of sense of gain, happiness, and sense of security differ, they are mutual and interdependent instead of isolated, forming an organic and unified whole. (1) Sense of gain is the basic source of happiness and sense of security. In the historical background of a new era, happiness and sense of security are only possible in the presence of a stable sense of gain. (2) Happiness is the highest expression of sense of gain and sense of security. Enhancing happiness is a global and systematic project. Individuals who maintain high levels of sense of gain and sense of security for a period of time experience high levels of happiness regarding the state of their real lives. (3) Sense of security is crucial for sense of gain and happiness. As a stable and peaceful feeling, sense of security is the prerequisite for perceiving and acquiring a sense of gain and happiness (Lu and Huan, 2017; Jin and Tao, 2018). Sense of gain, happiness, and sense of security are produced in the real context of daily livelihood and work, reflecting the principal contradictions of livelihood and work. Sense of gain, happiness, and sense of security have broad connotations, rigorous logic, and great significance, and improving them is critical to a decisive victory in establishing a moderately prosperous society and the realization of the Chinese dream of the great rejuvenation of the Chinese nation, which is a core objective of daily livelihood and construction in the new era (Jin and Tao, 2018).

Researchers have conducted studies on sense of gain, happiness, and sense of security, obtaining valuable results (e.g., Lu and Huan, 2017; Jin and Tao, 2018; Wang and Liu, 2019). However, several crucial topics require further empirical exploration: (1) the relationships among sense of gain, happiness, and sense of security in university students and (2) methods for improving university students' sense of gain, happiness, and sense of security. The university students, with higher levels of idealism and greater amounts of energy, constitute an important source of potential prosocial behavior agents, and their prosociality is very crucial in developing a community (Feng and Guo, 2017).

### Relationships Among Sense of Gain, Sense of Security, and Happiness

As society continues to develop, people are no longer limited to satisfying only their material needs. People have higher standards for happiness, and it has become a crucial topic among researchers and the general public. Happiness is an individual's overall assessment of objective life based on self-determined standards (Diener, 2012). Happiness is the core expression index of human best function. When the overall life is good, individuals will feel happy and good (Veenhoven, 2011). Individual's material satisfaction, spiritual needs, safety of life and property are all essential for happiness (Ma and Liu, 2017), and improving happiness is of substantial significance to the overall development of an individual.

Individuals differ in their approaches to pursue and attain happiness. Some people feel happy by pursuing sensory satisfaction and pleasure, whereas others feel happy by living a meaningful life (Guo et al., 2018). Hedonism and eudaimonia are two philosophical concepts concerning the realization of happiness. Hedonism refers to the pursuit of happiness through joy, comfort, and the satisfying of needs; that is, it is the sum of all happy moments (Ryan and Deci, 2001). In contrast to hedonism, eudaimonia refers to the pursuit of happiness through the continuous improvement of the self and the full realization of individual's potential through the pursuit of complex and meaningful goals. Happiness is attained by pursuing a purposeful and meaningful life and maximizing self-worth (Huta and Ryan, 2010). Peterson et al. (2005) proposed a model for authentic happiness based on hedonism and eudaimonia, suggesting that happiness includes not only enjoyment but also the establishment of meaningful goals and the engagement in pursuing of such goals. Authentic happiness more comprehensively reflects people's overall happiness; thus, this study divided happiness into pleasure happiness, meaning happiness, and engagement happiness to reflect overall happiness.

Since General Secretary Xi's proposal of “enabling the people to have a greater sense of gain,” sense of gain has become an increasingly crucial topic in social discussions and academic research. Sense of gain is a compound vocabulary comprising the objective, specific “fulfillment” and the subjective, abstract “sense”, and it is divided into objective and subjective levels. Several researchers have focused on the objective level of actual fulfillment. For example, Xing and Niu (2018) believed that sense of gain is a social development evaluation index oriented toward the basic needs and feedback of the people, indicating various specific forms of social welfare. Among researchers who have placed a greater emphasis on the subjective perception of sense of gain, Zhang (2016) suggested that sense of gain refers to the long-lasting satisfaction produced by material and spiritual fulfillment. Tan (2020) studied the relevant research paradigms of sociology and psychology for the exploration and empirical testing of the conceptual structure of sense of gain. He defined sense of gain as the comprehensive response of an individual's subjective cognition, emotional experience, and behavioral experience to the process and results of satisfying their needs in the development of social reform. Specifically, sense of gain is a subjective feeling formed on the premise of participation and contribution as well as the basis of objective acquisition. It is the integration of objective acquisition and subjective feeling. Objective acquisition not only includes interests in material and economic aspects but also rights to know, to participate, to express, to supervise, and the opportunity for self-actualization. Sense of gain, as a kind of subjective feeling, different individuals may have different subjective perceptions for the same objective acquisition (Gu et al., 2020).

Sense of gain is a subjective evaluation of one's benefits and a personal consciousness of internal satisfaction and pleasure. Sense of gain is closely related to happiness. If the pursuit of happiness is the ultimate value goal and end result of the development of human society, then sense of gain provides a realistic foundation and a feasible method for enhancing positive experiences and fully realizing national happiness (Xing and Niu, 2018). The enhancement of sense of gain is the foundation of the enhancement of happiness. An individual's happiness can only be improved if their need for a more favorable life is satisfied (Ma and Liu, 2017). The sense of gain is formed in the process of individuals' comparison with themselves and others, and its connotation corresponds to the existing concept “relative deprivation” (Zheng, 2020). Relative deprivation is that individuals or groups perceive that they are in a disadvantageous position through horizontal or vertical comparison with the reference group, and then experience some negative subjective cognitive and emotional experience (Mummendey et al., 1999). Once individuals or groups have relatively deprived psychological experience, it is very easy to form negative emotional and behavioral reactions (Smith et al., 2011).

Sense of security is the premonition of possible physical or psychological dangers or risks in terms of an individual's sense of power or powerlessness to cope with and manage them. Sense of security is primarily manifested in a sense of certainty and control (Cong and An, 2004). Wang and Liu (2019) reviewed 1,762 samples from a 2018 social mentality survey on sense of gain to investigate the relationships among sense of gain, sense of security, and happiness, discovering a significant correlation among sense of security, sense of gain, and happiness. Improving sense of security is the prerequisite for improving sense of gain and happiness. Only in a safe environment can people fully develop their potential, create more material and spiritual wealth, and experience the happiness they obtain. The safety of the internal and external environment and the resulting sense of security are essential for ensuring a sense of gain and happiness (Ma and Liu, 2017). Individuals with low sense of security often distrust others, often feel that they are not accepted, are prone to anxiety and show neuroticism. Niu et al. (2020) found that adolescents' psychological security was negatively correlated with their depression. Individuals with a higher sense of security are more receptive to themselves and trust others. They can experience more sense of belonging and control and will not consider themselves a burden to others (Yuan et al., 2020). These individuals with a high sense of security can usually maintain a stable and positive intimate relationship with others, less experience the feeling of being isolated and abandoned by the world, and less experience the sense of incompetence (Yuan et al., 2020).

To date, there has been a scarcity of empirical literature that measures sense of gain, sense of security, and happiness at the same time, and there is still a scarcity of empirical literature focusing on the relationships among the three. We only found one empirical study conducted in mainland China, which explored the relationships among sense of gain, sense of security, and happiness (Wang and Liu, 2019). By correlation analysis, the researchers found that sense of gain, sense of security, and happiness are interrelated. Thus, sense of gain, sense of security, and happiness are closely related. Sense of gain is the foundation for happiness, and sense of security guarantees the enhancement of happiness. Accordingly, this study inferred that sense of security may have a moderating effect on the relationship between sense of gain and happiness; that is, the higher the sense of security, the greater the effect of the sense of gain on happiness.

### Methods for Enhancing Sense of Gain, Sense of Security, and Happiness on the Basis of Prosocial Behaviors

The 19th National Congress of the Communist Party of China proposed to “keep up with people's ever-growing needs for a better life. We will continue to promote social fairness and justice, develop effective social governance, and maintain public order. With this we should see that our people will always have a strong sense of gain, happiness, and sense of security.” Approaches to enhancing sense of gain, sense of security, and happiness in a new era are worthy of research.

Prosocial behaviors represent a broad category of actions defined by a significant segment of society or social group as being generally beneficial to other people (Penner et al., 2005). Such behaviors may appear selfless, but they can provide internal feedback to individuals both intentionally and unintentionally. In interpersonal relationships, prosociality helps promote communication, adaptation, and harmony (Campbell et al., 1999). From individuals perspective, prosociality enhances self-esteem and self-satisfaction (Laible et al., 2004). As the saying goes, “what goes around comes around;” that is, prosocial behavior is not only a means for the one-way transfer of resources to the recipient of the behavior but also a self-motivation process for the performer (Xie et al., 2017). Performing prosocial behaviors can provide more meaning and efficacy to the performer (Sonnentag and Grant, 2012), and engaging in prosocial behaviors enables individuals to more effectively cope with psychological pressure (Li and Ferraro, 2005). Dillon and Wink (2007) found that individuals who show more prosocial and helpful behaviors during their youth are more physically and mentally healthy in late adulthood. Dunn et al. (2008) found that participants who spend money on others experienced greater happiness than those spend money on themselves, specifically, both cross-sectionally in a nationally representative survey study and longitudinally in a field study of windfall spending, spending more of one's income on others predicted greater happiness. Weinstein and Ryan (2010) found in a Western cultural context that prosocial behaviors with autonomous motivation are beneficial for happiness. In collectivistic cultural background such as China, prosocial behaviors may be performed as a matter of role or obligation and be activated mainly by extrinsic motivation, not functioning as a means of fulfilling autonomy (Smith et al., 2013). We investigated these relationships in Chinese cultural setting to test whether positive effects exist by measuring types of happiness. Moreover, Martela and Ryan (2016) found that prosocial behaviors increased well-being even without contact with the beneficiary, and the relationship was mediated by autonomy and competence needs satisfaction. Hui and Kogan (2018) found that prosocial engagement promotes state well-being, with state competence need satisfaction acting as both the moderator and mediator of this link. The aforementioned empirical studies demonstrate that prosocial behaviors are beneficial to individuals' mental health and can enable them to be happier and have greater life satisfaction.

Sense of gain includes the satisfaction at both the material and spiritual level (Zhang, 2016). Under modern conditions, material living standards have greatly improved, and people have greater satisfaction at the material level (Wen and Liu, 2018). After their material needs are met, people will actively pursue spiritual satisfaction, and prosocial behaviors can help them attain a sense of self-worth and social value (Hao, 2019). Studies have demonstrated that actively providing others with the appropriate assistance within one's power does not constitute a personal loss; rather, it considerably improves the happiness and satisfaction of the helper (Yang and Kou, 2017). Motivation to perform prosocial behaviors involves both purely altruistic motivation for the benefit of others as well as self-service motivation to enhance individuals' self-worth and social value (Zhang and Kou, 2012). When individuals help others and benefit society, they not only receive the gratitude of others and the appreciation of society but also acquire direct satisfaction.

Prosocial values represent a virtuous attitude that is advocated and respected in pursuit of constructing a harmonious society. If the conclusion that prosocial behaviors improve the sense of gain, sense of security, and happiness of university students in the new era can be confirmed in the context of Chinese culture, university students can be motivated to perform more prosocial behaviors, which will help establish a harmonious, mutually beneficial, and positive social atmosphere. Accordingly, this study explored methods for enhancing sense of gain, sense of security, and happiness on the basis of prosocial behaviors. This study assumed that sense of gain plays mediating roles between prosocial behaviors and the three types of happiness and that the aforementioned mediating roles were moderated by sense of security.

## Materials and Methods

### Participants

A power analysis was conducted using G^*^Power 3.1 (Faul et al., 2007) in which the estimated effect size of *r* = 0.13, α = 0.05 (two-tailed), and power = 0.80. The result suggested a required sample size of *N* = 362. Thus, we chose to examine the associations of prosocial behaviors with happiness in a larger sample to increase sensitivity. A total of 680 university students were recruited from more than ten faculties at a university, located in Shandong Province, Eastern China, and 680 questionnaires were distributed. After invalid questionnaires were excluded because there were missing data on key study variables, a total of 646 valid questionnaires remained, with a valid response rate of 95.00%. The participants consisted of 274 men (42.41%) and 372 women (57.59%) aged 18–24 years with an average age of 20.52 years, with a standard deviation of 2.19.

About their demographic information, 28.95% (187) and 71.05% (459) of the participants were only children or had siblings, respectively. Of the participants, 25.08% (162), 18.11% (117), and 56.81% (367) were from city, township or countryside, respectively. Participants from nuclear families (living with parents), multigenerational families (living with paternal or maternal grandparents and parents), single-parent families (living with only father or mother), blended families (living with father and stepmother or living with mother and stepfather), no-parents families (not living with any parents) accounted for 78.02% (504), 17.03% (110), 2.78% (18), 2.17% (14), and 0% (0), respectively. The detailed results were summarized in Table 1.

**Table 1:** Demographic characteristics information.

**Variables**	**Frequency**	**Percent**	**Minimum**	**Maximum**	***M***	***SD***
Gender			1	2	1.58	0.50
Men	274	42.41%				
Women	372	57.59%				
Only children			1	2	1.71	0.45
Yes	187	28.95%				
No	459	71.05%				
Home location			1	3	2.32	0.85
City	162	25.08%				
Township	117	18.11%				
Countryside	367	56.81%				
Family composition			1	5	1.29	0.63
Nuclear families	504	78.02%				
Multigenerational families	110	17.03%				
Single-parent families	18	2.78%				
Blended families	14	2.17%				
No-parents families	0	0%				

### Procedure

In this cross-sectional study by means of an online questionnaire survey, convenience cluster sampling was used. All data were gathered during summer 2020. We contacted university students through the internet and communicated with them. After obtaining their informed consent, we sent online questionnaire and they conducted the questionnaire survey online. These participants are willing to cooperate with this survey and can independently complete the questionnaires. We did not send questionnaires to those who were not willing to cooperate with the investigation. Participants were told to answer all the questions accurately and truthfully based on their feelings and experiences in their daily lives. They completed the Demographic Questionnaire, Sense of Gain Scale, Sense of Security Scale, Orientations to Happiness Scale, and Prosocial Behavior Tendencies Scale (total 93 items). The measures were administrated to the participants by two trained research assistants online. The data collection took about 35 min. At the end of the study, each participant received a bonus (RMB 8 = US $1.23) as compensation.

### Ethical Statements

This study was conducted under the approval and direction of the Institutional Review Board (IRB) at the University and was conducted in accordance with the Helsinki declaration. The participants were made aware of the voluntary and confidential nature of this study. They were fully informed of the research before participation, such as purpose and content. Written consent was obtained prior to the administration. All participants were over 18 years old, and there were no minors involved. This study caused no harm to participants' physical and mental health, and the results of this study were maintained confidentially.

### Measures

#### Demographic Questionnaire

The participants provided demographic characteristics information including their age, gender, and the number of children in their home, their home location, and family composition. About the family composition, Bengtson (2001) suggested that family multigenerational relations become more important, in reply to the widely debated “family decline” hypothesis, which assumes a nuclear family model of two biological parents and children. In research of Chiu (2007), family variables included living with two-birth parents, single parent, blended family, and no parents. Cheng et al. (2017) categorized family structure as nuclear family or extended family. Based on these previous research, in this study, the family composition included five types: nuclear families (living with parents), multigenerational families (living with paternal or maternal grandparents and parents), single-parent families (living with only father or mother), blended families (living with father and stepmother or living with mother and stepfather), and no-parents families (not living with any parents).

#### Sense of Gain Scale

The Sense of Gain Scale compiled by Dong et al. (2019) was used to measure university students' sense of gain. It reflects the individual's cognitive evaluation of the content, realization ways and required conditions for obtaining the satisfaction of their own needs, as well as the psychological experience in this process (Dong et al., 2019). The scale comprises 28 items in five dimensions: (1) gaining experience (six items; e.g., “My current life is comfortable”); (2) gaining environment (six items; e.g., “Open social policies have provided a new aspect to my life”); (3) gaining content (six items; e.g., “I care about others' trust in me”); (4) gaining way (five items; e.g., “I will do my best to achieve my life goals”); and (5) gaining sharing (five items; e.g., “I sincerely praise those who have contributed to society”). A seven-point Likert scale ranging from 1 (completely disagree) to 7 (completely agree) was adopted, with a higher score indicating a higher sense of gain. The Chinese version of this scale has been established to apply to Chinese population (Dong et al., 2019), and it has been validated in the Chinese population (Tan, 2021). In this study, the α coefficient of the overall Sense of Gain Scale was 0.96, and the α coefficients for the five dimensions were 0.96, 0.95, 0.90, 0.93, and 0.93, respectively.

#### Sense of Security Scale

The Sense of Security Scale compiled by Cong and An (2004) was employed to measure university students' sense of security. The scale comprises 16 items in two dimensions: (1) sense of interpersonal security (eight items; e.g., “I never dare to take the initiative to express my opinion”) and (2) sense of certain control (eight items; e.g., “I feel that life is always uncertain and unpredictable”). A five-point Likert scale ranging from 1 (very inconsistent) to 5 (very consistent) was adopted, with a higher score indicating a lower sense of security. The Chinese version of this scale has been established to apply to the Chinese people (Cong and An, 2004), and it was validated in China and displayed satisfactory reliability and validity (Zhang and Xu, 2020). In this study, the α coefficient of the overall Sense of Security Scale was 0.96, and the α coefficients for the two dimensions were 0.92 and 0.93, respectively.

#### Orientations to Happiness Scale

The Orientations to Happiness Scale revised by Chen (2010) was used to measure university students' happiness. The scale comprises 18 items in three dimensions: (1) meaning happiness (six items; e.g., “My life has a greater purpose”); (2) pleasure happiness (six items; e.g., “Life is short, so you must live your life to the fullest”); and (3) engagement happiness (six items; e.g., “No matter what I am doing, I always feel that time passes quickly”). A five-point Likert scale ranging from 1 (very unlike me) to 5 (very like me) was adopted, with a higher score implying higher individual's happiness. The Chinese version of this scale has been established to apply to Chinese university students (Chen, 2010), and it was found to have appropriate psychometric properties for the Chinese undergraduates (Zhou, 2013). In this study, the α coefficient of the overall Orientations to Happiness Scale was 0.92, and the α coefficients for the three dimensions were 0.86, 0.82, and 0.85, respectively.

#### Prosocial Behavior Tendencies Scale

The Prosocial Behavior Tendencies Scale revised by Kou et al. (2007) was used to measure university students' prosocial behaviors. The scale comprises 26 items in six dimensions: (1) public prosocial behaviors (four items; e.g., “I do my best to help others when someone is present”); (2) anonymous prosocial behaviors (five items; e.g., “I prefer to donate anonymously”); (3) altruistic prosocial behaviors (four items; e.g., “I do not donate money or materials for my own benefit”); (4) compliant prosocial behaviors (five items; e.g., “I rarely refuse when others ask me for help”); (5) emotional prosocial behaviors (five items; e.g., “I feel very good when I can comfort someone in a bad mood”); and (6) dire prosocial behaviors (three items; e.g., “I tend to help those who are really in trouble and urgently need help”). A five-point Likert scale ranging from 1 (very unlike me) to 5 (very like me) was employed, with a higher score indicating a strong orientation to prosocial behaviors. The Chinese version of this scale has been established to apply to Chinese middle school and university students (Kou et al., 2007), and it was found to have appropriate properties for the Chinese people (Feng and Zhang, 2021). In this study, the α coefficient of the overall Prosocial Behavior Tendencies Scale was 0.96, and the α coefficients of the six dimensions were 0.85, 0.87, 0.86, 0.86, 0.88, and 0.81, respectively.

### Statistical Analysis

Correlation analysis in this study was conducted with the statistical software SPSS 19.0. We adopted PROCESS macro Model 14 to conduct a moderated mediation analysis (Hayes, 2013) to examine the effects of prosocial behaviors on happiness, mediated by sense of gain and moderated by sense of security. In addition, Amos 23.0 was used to conduct path analysis with maximum likelihood estimation to examine the mediating effects of sense of gain between prosocial behaviors and the three types of happiness. The significance limit was set at *p* < 0.05. Evaluations of structural equation modeling (SEM) models were conventionally based on the following statistics: the normed fit index (NFI), the relative fit index (RFI), the incremental fit index (IFI), the comparative fit index (CFI), the Tucker-Lewis index (TLI), and the root-mean-square error of approximation (RMSEA) (McDonald and Ho, 2002; Kline, 2005).

## Results

### Common Method Bias Test

Because the data were collected from university students by using self-report questionnaires, common method bias was a possibility. Accordingly, the following approach was adopted to cope and handle common method bias. First, procedural control was adopted. While conducting the survey, anonymity and confidentiality were emphasized, and the researcher explained to the participants that the data would only be used for scientific research to ensure that the collected data would be reliable (Zheng and Wang, 2017). In addition, all measurement items met the criteria for avoiding ambiguity (e.g., ambiguous sentences and complex academic jargon), and the disclosure of subjective attitudes was avoided (Tu et al., 2017). Finally, statistical control was implemented, and Harman's single factor test was conducted to perform factor analysis on all questionnaire items to identify any common method bias (Zhou and Long, 2004). The results indicated that 12 factors exhibited eigenvalues >1 without rotation, and the variance explained was 28.28%, which was less than the critical standard of 40%, implying that common method bias was not evident.

### Descriptive Statistics and Correlation Analysis

Pearson correlation analysis was conducted for variables including prosocial behaviors, sense of gain, sense of security, and happiness (including meaning, pleasure, and engagement happiness). The results (Table 2) demonstrate that prosocial behaviors were significantly and positively correlated with sense of gain and the three types of happiness (*p* < 0.001); however, prosocial behaviors were not significantly correlated with sense of security (*p* > 0.05). Sense of gain had a significant negative correlation with sense of security (*p* < 0.001) and significant positive correlations with the three types of happiness (*p* < 0.001). Finally, sense of security was not significantly correlated with the three types of happiness (*p* > 0.05).

**Table 2:** Correlations among the key study variables (*N* = 646).

	**1**	**2**	**3**	**4**	**5**	**6**
1 Prosocial behaviors	–					
2 Sense of gain	0.54^***^	–				
3 Sense of security	0.03	−0.14^***^	–			
4 Meaning happiness	0.49^***^	0.61^***^	−0.06	–		
5 Pleasure happiness	0.39^***^	0.45^***^	0.05	0.60^***^	–	
6 Engagement happiness	0.40^***^	0.50^***^	−0.03	0.76^***^	0.62^***^	–
*M*	3.88	5.83	2.80	3.84	3.73	3.63
*SD*	0.63	0.81	0.95	0.66	0.69	0.67

*** *p < 0.001*.

### Relationships Between Prosocial Behaviors and Happiness: The Moderated Mediation Analysis

Prosocial behaviors, sense of gain, and sense of security were used as the independent, mediating, and moderating variables, respectively, whereas meaning, pleasure, and engagement happiness were used as the dependent variables. The regression analysis results (Table 3) indicated that prosocial behaviors significantly and positively predicted sense of gain (β = 0.69, *p* < 0.001), meaning happiness (β = 0.24, *p* < 0.001), pleasure happiness (β = 0.20, *p* < 0.001), and engagement happiness (β = 0.19, *p* < 0.001). Sense of gain significantly and positively predicted meaning happiness (β = 0.41, *p* < 0.001), pleasure happiness (β = 0.31, *p* < 0.001), and engagement happiness (β = 0.34, *p* < 0.001). Furthermore, sense of security did not significantly predict meaning, pleasure or engagement happiness (*p* > 0.05). Moreover, the interaction between sense of security and sense of gain had no significant effect on the prediction of the three types of happiness (*p* > 0.05).

**Table 3:** Regression analysis of moderated mediation effect.

**Regression equation (*****N*** **=** **646)**		**Fitting index**			**Coefficient significance**	
**Dependent variable**	**Independent variable**	***R***	***R^**2**^***	***F***	*****β*****	***t***
Sense of gain		0.54	0.29	269.35^***^		
	Prosocial behaviors				0.69	16.41^***^
Meaning happiness		0.65	0.42	114.23^***^		
	Prosocial behaviors				0.24	6.36^***^
	Sense of gain				0.41	13.52^***^
	Sense of security				−0.01	−0.54
	Sense of security × Sense of gain				0.04	1.89
Pleasure happiness		0.49	0.24	50.84^***^		
	Prosocial behaviors				0.20	4.29^***^
	Sense of gain				0.31	8.69^***^
	Sense of security				0.07	2.70
	Sense of security × Sense of gain				−0.03	−1.02
Engagement happiness		0.53	0.28	62.03^***^		
	Prosocial behaviors				0.19	4.32^***^
	Sense of gain				0.34	10.02^***^
	Sense of security				0.02	0.79
	Sense of security × Sense of gain				−0.02	−0.90

*** *p < 0.001*.

In addition, the percentile bootstrap (bootstrap samples: 5,000; confidence interval: 95%) was used, and the SPSS PROCESS macro program Model 14 compiled by Hayes (2013) was employed to examine the mediating effects of sense of gain on the relationships between prosocial behaviors and happiness and to determine whether the mediating effects were moderated by sense of security. The 95% bootstrap confidence interval of the mediating effects of sense of gain on the relationships between prosocial behaviors and meaning, pleasure, and engagement happiness did not contain 0 (Table 4). The mediating effects of sense of gain on the relationships between prosocial behaviors and meaning, pleasure, and engagement happiness accounted for 54.00, 49.00, and 55.00% of the total effect, respectively. Therefore, sense of gain played mediating roles between prosocial behaviors and happiness; moreover, the mediating roles were not moderated by sense of security.

**Table 4:** Bootstrap analysis of moderated mediation effect.

	**Effect value**	**Boot standard error**	**Boot CI**	**Boot CI**	**Effect ratio %**
			**Lower limit**	**Upper limit**	
Prosocial behaviors → Meaning happiness	0.24	0.04	0.16	0.31	46.00
Prosocial behaviors → Pleasure happiness	0.22	0.04	0.13	0.30	51.00
Prosocial behaviors → Engagement happiness	0.19	0.04	0.11	0.28	45.00
Prosocial behaviors → Sense of gain → Meaning happiness	0.28	0.04	0.21	0.36	54.00
Prosocial behaviors → Sense of gain → Pleasure happiness	0.21	0.04	0.14	0.28	49.00
Prosocial behaviors → Sense of gain → Engagement happiness	0.23	0.03	0.17	0.30	55.00

**Figure 1:**
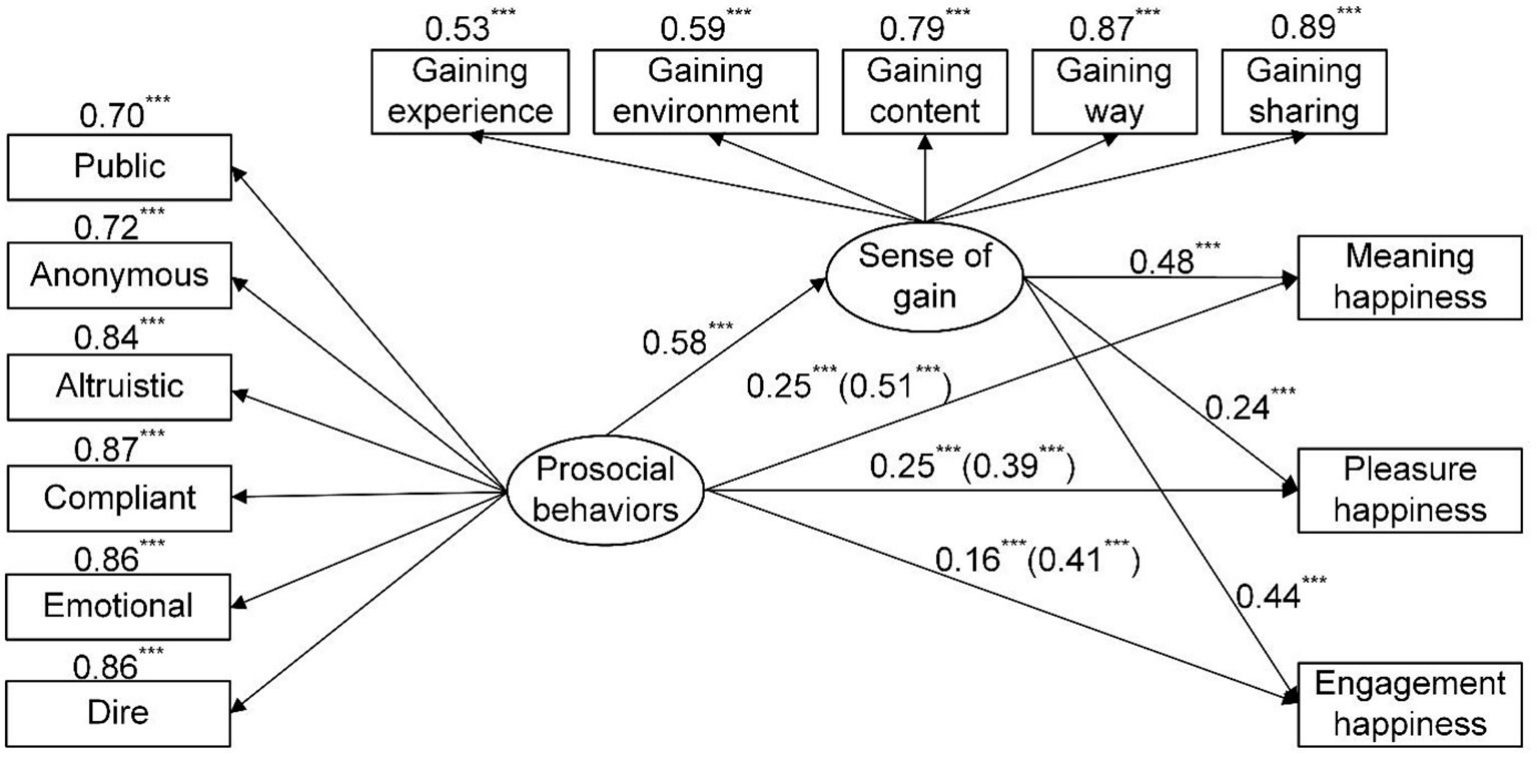
Relationship model and standardized path coefficients of prosocial behaviors, sense of gain and three types of happiness. ***p < 0.001.

Because sense of security did not significantly moderate the mediating role of sense of gain in the relationships between prosocial behaviors and happiness, this study further investigated the mediating roles of sense of gain in the relationships between prosocial behaviors and happiness through structural equation modeling (SEM). Prosocial behaviors and sense of gain were used as independent and mediating variables, respectively, whereas meaning, pleasure, and engagement happiness were adopted as the dependent variables. A structural equation model (Figure 1) was constructed to examine the mediating roles of sense of gain in the relationships between prosocial behaviors and happiness. Overall, the model exhibited favorable goodness of fit (χ^2^ = 310.836, *df* = 63, χ^2^/*df* = 4.934, RMSEA = 0.078, NFI = 0.952, RFI = 0.931, IFI = 0.962, CFI = 0.961, TLI = 0.944). Specifically, prosocial behaviors significantly and positively predicted sense of gain and meaning, pleasure, and engagement happiness, and sense of gain further significantly and positively predicted meaning, pleasure, and engagement happiness, indicating that sense of gain had significant mediating effects on the relationships between prosocial behaviors and happiness. The explanatory rates of the model for meaning, pleasure, and engagement happiness were 43.00, 18.90, and 30.30%, respectively.

## Discussion

### Relationships Among Sense of Gain, Sense of Security, and Happiness

This study examined the relationships among sense of gain, sense of security, and happiness. First, the results revealed that sense of gain significantly predicted happiness, this is similar to proposal of Ma and Liu (2017) as well as Xing and Niu (2018) who proposed that sense of gain is closely related to happiness, and the improvement of sense of gain is the basis for the improvement of happiness. Sense of gain refers to the sense of satisfaction after certain benefits are obtained, and happiness is the subjective feeling of satisfaction with life. Thus, sense of gain is the foundation of happiness, whereas happiness is the extension of sense of gain. Because of its ease of transformation into happiness, the realization of sense of gain indicates the enhancement of happiness (Kang, 2016; Wang and Liu, 2019). The prerequisite for generating happiness is the possession of sense of gain, without which happiness is empty and abstract. Happiness originates from the subjective perception and emotional sublimation of self-satisfaction. The enhancement of sense of gain is a means for improving happiness. The acquisition of individual's interests, having actual needs met, security of lives and property are all pursued to enhance happiness. Only when individuals benefit from social development, their actual needs are met, and their lives and property are secure, can they lay a solid foundation for enhancing happiness (Ma and Liu, 2017). The sense of gain is more specific and situational, in contrast, the happiness is more sustainable and meaningful (Tan et al., 2020a). The sense of gain based on the satisfaction of needs is the basis for people to be satisfied with life and improve their happiness. Therefore, to a certain extent, the happiness can be understood as the accumulated sense of gain. The continuous sense of gain will lead to the improvement of the happiness and is the guarantee of the continuous happiness (Wang and Liu, 2019).

About the moderating effect of sense of security, sense of security did not have a moderating effect on the relationships between sense of gain and happiness. Zheng (2020) also found that whether it is low or high sense of security group, Chinese citizens' sense of gain has significant positive impacts on happiness. The Chinese public has a relatively high sense of security, with most of them holding a positive attitude toward life, production, emotional safety, and future expectations. In the 2020 World Law and Order Index, China ranked third, with a score of 94, indicating that Chinese people have a high sense of security. While the World Happiness Report 2020 indicated that China ranked 94th among the 156 surveyed countries. Thus, the Chinese people generally have a high sense of security but a relatively low level of happiness. This may be because happiness is derived more from people's subjective feelings. Although happiness is established on the foundation of sense of gain and sense of security, it is also affected by various complex factors such as equal opportunities and social justice (He and Pan, 2011; Ni, 2020). This is also a crucial reason that sense of security did not play a moderating role between sense of gain and happiness. Thus, when enhancing sense of gain to rapidly improve happiness, considering the “catalyst” of sense of gain to generate different aspects of happiness is necessary. About the relationships between sense of security and happiness, this study found that the relationships between sense of security and three kinds of happiness were not significant, and the results were dissimilar to Wang and Liu (2019) who found that sense of security and sense of happiness were significantly correlated. The reasons may be as follows: Wang and Liu (2019) conducted the survey on sense of security drawing on the classification of Vail (1999), that is, the sense of security is divided into personal safety, property safety, traffic safety, medical safety, food safety, labor safety, and personal information privacy safety as well as environmental safety. While in this study, the sense of security is divided into sense of interpersonal security and sense of certain control, it is an individual's feeling of uncertainty and insecurity in a certain social environment.

### Methods for Enhancing of Sense of Gain, Sense of Security, and Happiness on the Basis of Prosocial Behaviors

In this study, the implementation of prosocial behaviors was used as a method for improvement, specifically in terms of sense of gain, sense of security, and happiness. Because sense of security was discovered to lack a moderating role in the relationships between sense of gain and happiness in the aforementioned discussion, only the relationships between prosocial behaviors and sense of gain and happiness were explored. The results revealed that prosocial behaviors significantly predicted sense of gain and happiness and that sense of gain significantly predicted happiness. Thus, sense of gain had mediating effects on the relationships between prosocial behaviors and happiness; that is, prosocial behaviors had a direct influence on happiness and an indirect influence on happiness through sense of gain.

Notably, in the separate exploration of meaning, pleasure, and engagement happiness, prosocial behaviors had significant impacts on all three types of happiness. This is similar to previous research of Feng (2018) who concluded that prosocial behaviors can positively influence well-being under Chinese cultural background. However, the researcher did not pay attention to different types of well-being. Compared with simply “getting,” active participation in “giving” activities such as prosocial behaviors can make people get more lasting happy experience (O'Brien and Kassirer, 2019). Prosocial behaviors enable individuals to experience more positive emotions and fewer negative emotions (Gebauer et al., 2008). The positive emotion effect explained that people are willing to help others because prosocial behaviors enable them to maintain a favorable mood and have a more positive experience (Zhu et al., 2010). Moreover, according to the negative-state relief model proposed by Baumann et al. (1981), helping others, which can reduce anxiety and depression, is an effective method for reducing negative emotions. In this sense, prosocial behaviors help individuals pursue sensory satisfaction and pleasure, thereby helping them attain happiness. The eudemonistic view of happiness suggests that meaningful activities can provide people with pleasant experiences. Individuals focusing on meaning and engagement happiness obtain happiness by engaging in activities that help them realize their potential and bring meaning to their lives, helping them to fulfill their potential (Huta et al., 2012; Carsten et al., 2015). We also found that the predictions of prosocial behaviors on meaning and engagement happiness were stronger than that of prosocial behaviors on pleasure well-being. Previous studies have suggested that meaning and engagement contribute more to individuals' well-being than pleasure (e.g., Kavčič and Avsec, 2014; Avsec et al., 2016). Prosocial behaviors can help individuals find meaning in their lives. They can establish closer relationships with others through prosocial behaviors, such as helping others and donating money, thereby finding meaning in life that transcends themselves (Van Tongeren et al., 2015) and facilitating the continuous improvement of self-worth, which helps them to acquire more meaning and engagement happiness.

Sense of gain had mediating effects on the relationships between prosocial behaviors and happiness. About the mediating effects of sense of gain, there is no similar previous studies. To a large extent, the enhancement of sense of gain depends on the continuous satisfying of personal needs. People instinctively pursue a sense of material gain for basic satisfaction, but pursuit of a sense of spiritual gain represents higher aspirations (Ou and Su, 2018). Thus, the satisfying of spiritual needs is rarer than that of material needs. Maslow's hierarchy of needs includes both low-level physiological needs and high-level spiritual and psychological needs, with the highest-level needs being growth needs. Such needs are not governed by instinct but are driven by the fulfillment of one's potential to achieve higher levels of satisfaction (Maslow, 1981; Tan et al., 2020b). Prosocial behaviors can meet such high-level needs as well as the needs of individuals to find meaning in life and achieving prosperity (Yang and Kou, 2015). Relevant studies have revealed that prosocial spending can improve the provider's happiness (e.g., Diener and Seligman, 2004; Kuykendall et al., 2015). Notably, such happiness is more evident when one or more core needs can be met (Dunn et al., 2014). The process of helping others is also a process of proving one's own usefulness (Son and Wilson, 2012). Prosocial behaviors enable recipients to meet their own needs while receiving assistance, and they promote favorable relationships with the provider, thereby enabling the provider to obtain the spiritual satisfaction required to affirm self-worth. Sense of gain is based on the satisfying of psychological needs, which are the source of individual's happiness (Deci and Ryan, 2000). Happiness is improved when certain behaviors can satisfy their psychological needs (Ryan and Deci, 2000). Prosocial behaviors can feasibly achieve the satisfying of individual's psychological needs, thereby enhancing happiness (Feng, 2018).

### Limitations

There are also some limitations in this study that should be borne in mind when assessing the value of the findings. First, the correlational design used in this study weakens inference about the causal relationships between prosocial behaviors and happiness. Future research is needed to specify the direction of the relations with a longitudinal method that investigates the lag effect of prosocial behaviors on happiness or experimentally manipulates prosociality to explore the resultant change in happiness. Second, participants in this study might be relatively homogenous (from only one province in Eastern China). So the findings should be treated with caution. Future research is needed to replicate these results in more representative samples. Third, we mainly relied on self-reports, which may lead to reliance on self-awareness and reported biases (Stone et al., 2000). Future researchers could test the effect of prosociality with more varied procedures.

## Conclusion and Implications

This study revealed that sense of gain significantly and positively predicted happiness. However, sense of security and the interaction between sense of security and sense of gain did not significantly predict happiness; therefore, sense of security did not have a moderating effect on the relationships between sense of gain and happiness. Prosocial behaviors significantly and positively predicted sense of gain and happiness, and sense of gain played mediating roles in the relationships between prosocial behaviors and happiness. In summary, the findings present certain implications for improving university students' sense of gain, sense of security, and happiness, specifically by promoting prosocial behaviors. University is a critical period of growth, and the attainment, understanding, and judgment of sense of gain, sense of security, and happiness will have a crucial impact on physical and mental health of students and on the formation of their outlook on the world, life, and values. Despite the importance of improving university students' happiness, appropriate measures should be implemented instead of focusing only on the satisfying of material needs, the pursuit of pleasure, and short-term emotional satisfaction. Spiritual gain and the pursuit of psychological needs should also be emphasized. This study revealed that prosocial behaviors have a positive impact on the improvement of university students' happiness. Prosocial behaviors are a continuum ranging from self-benefit to altruism, and prosocial behaviors have both personal significance and social value. Therefore, promoting the cultivation and development of prosocial behaviors in university students can facilitate their integration into society as well as their contribution to others and society. It can also help them improve their sense of gain and happiness, enabling them to form appropriate values, satisfy their spiritual and cultural needs, and promote healthy and comprehensive development. When educators cultivate university students' prosocial behaviors, they should adopt additional practical teaching approaches to enable them to experience the benefits of prosocial behaviors. When students understand the benefits of such behaviors, it will prompt them to adopt prosocial behaviors, thereby internalizing the values and externalizing them in their actions.

This study explored the relationships among sense of gain, sense of security, and happiness as well as methods for improving them on the basis of prosocial behaviors. However, the findings did not fully meet the expectations of the research. Because of the relatively limited research on sense of security, which is crucial for individuals and necessary for maintaining mental health, future studies should further employ sense of security as a predicting variable to explore the impact of sense of security on individuals' sense of gain and happiness.

## Data Availability Statement

The raw data supporting the conclusions of this article will be made available by the authors, without undue reservation.

## Ethics Statement

The study involving human participants were reviewed and approved by Institutional Review Board (IRB) at Shandong University of Technology. The participants provided their written informed consent to participate in this study.

## Author Contributions

LF: conceptualization, funding acquisition, and writing—review & editing. LF and HZ: formal analysis, investigation, methodology, and writing—original draft.

## Funding

This research was supported by the Humanities and Social Sciences Project of Ministry of Education in China (18YJC190003).

## Conflict of Interest

The authors declare that the research was conducted in the absence of any commercial or financial relationships that could be construed as a potential conflict of interest.

## Publisher's Note

All claims expressed in this article are solely those of the authors and do not necessarily represent those of their affiliated organizations, or those of the publisher, the editors and the reviewers. Any product that may be evaluated in this article, or claim that may be made by its manufacturer, is not guaranteed or endorsed by the publisher.
